# Membrane Distillation Process

**DOI:** 10.3390/membranes11020144

**Published:** 2021-02-18

**Authors:** Alessandra Criscuoli

**Affiliations:** Institute on Membrane Technology (CNR-ITM), via P. Bucci 17/C, 87036 Rende (CS), Italy; a.criscuoli@itm.cnr.it

The water stress that we have been experiencing in the last few years is driving the development of new technologies for the purification and recovery of water. Membrane Distillation (MD) is based on the use of hydrophobic microporous membranes that prevent the passage of aqueous feed as a liquid through the micropores, allowing the transport of water vapor and volatiles only, thanks to a difference in partial pressures established across the membrane. In this way, high-purity distillates can be produced, starting from a variety of aqueous streams, like effluents coming from the textile/agrofood/pharmaceutical industry, olive mill wastewaters, waters contaminated by heavy metals, sea, and brackish waters. Some studies on the application of MD for the purification of radioactive wastewaters and of urine have also been carried out.

With respect to Reverse Osmosis (RO), which is limited by the osmotic pressure and sometimes shows low rejection values for elements like As(III) and Boron, MD is able to produce fresh water from high-concentrated streams and provides 100% theoretical rejections for all non-volatiles present in the aqueous feeds.

Despite these advantages, MD is far from a significant application at industrial scale, due to some pending issues:

- The need to develop membranes with high hydrophobicity and liquid entry pressure values, which can remain stable when treating real streams in long-term runs;
- The need to develop modules with reduced thermal and mass transfer resistances;
- The need to reduce the specific thermal energy consumption.

Research on the above-mentioned points is in progress, including the use of renewable energies to cover the thermal demand of the system and the integration of MD with other membrane units, in order to improve the overall performance of the processes.

The aim of this Special Issue is to provide an overview of the latest results obtained in the field for overcoming MD drawbacks and boosting its implementation at a large scale. Research efforts into the development of new membranes and modules, analysis of the MD performance for bio-feeds at relatively low temperatures, integration of MD with other membrane units and evaluation of MD at pilot scale are presented and discussed.

The mechanical resistance of a PVDF membrane was enhanced by preparing three-layered membranes by electrospinning the PVDF nanofibers on both sides of commercial PES nanofiber-based nonwoven supports [1]. Prepared membranes were first characterized and then tested in Direct Contact Membrane Distillation (DCMD) for the treatment of a 5 M NaCl feed. During 380’ of the test, complete salt rejections were registered, together with trans-membrane fluxes higher than the commercial PVDF membrane, due to the higher porosity of the produced composite membranes.

The mechanical properties and stability during DCMD tests of capillary PP membranes containing talc was investigated by Gryta [2]. Both PP and PP-containing-talc membranes were characterised and tested. The presence of talc led to an increase in porosity and enhanced the mechanical properties due to a disorder of the linear structure of PP. Distillation experiments were carried out on a 1 g/L NaCl feed for 350 h and both membranes led to constant flux and complete rejections. However, process efficiencies higher more than 10% were obtained with the talc addition. After tests, no talc leaching from the membrane was observed.

Composite membranes with hydrophobic coatings on hydrophilic supports were developed to enhance the trans-membrane flux in direct contact membrane distillation tests on salty solutions [3]. The use of hydrophilic porous polyethersulfone as a support led to a reduction in the gas gap for the water vapor transport and, therefore, at 70 °C, fluxes ranging from 27 to 18 kg/m^2^h were obtained for feed of 1 wt% and 10 wt% NaCl, respectively. The salt passage in the permeate was quite low for the more diluted feed (1 wt% NaCl), while it increased at higher salt content, indicating the need for longer time and/or a higher power supply for the coating step.

A crucial aspect in the design of membrane distillation modules is the choice of spacers, as they are a means to increase mixing of the involved streams, with improvements in heat transfer, but also a cause of pressure drops and reduced membrane area available for evaporation (when the spacers contact the membrane surface). Novel protopypes of overlapped spacers were designed and their performance was investigated by CFD simulations [4]. Spacers differed in terms of geometrical configuration (intrinsic angle between filaments and flow attack angle values). The simulation led to the calculation of the heat transfer coefficient as a function of the Reynolds number (varied from 200 to 800) and to the identification of the optimal geometrical configuration.

An important parameter to improve in view of a large-scale implementation of MD, is the water recovery ratio achievable in a single pass. In fact, low recovery ratios per pass are usually obtained in MD modules, and feed re-circulation or more MD modules working in series are needed to produce a reasonable quantity of distillate and to concentrate the feed up to desired values. A new module design, called the feed gap air gap MD (FGAGMD), was produced in plate and frame geometry, and tested with both tap water and NaCl feeds (117–214 g NaCl/kg) [5]. In the module, the feed was heated through a polymer film in contact with a heating solution, while the distillate condensed on another polymer film in contact with the coolant. If compared with an AGMD spiral wound module, the new module led to a significant improvement in the recovery ratio, with the other performance indicators being similar.

Most of the modules used in MD have an external housing and are characterized by the re-circulation of the feed to be treated. This means the need for a pump for re-circulation and a heater for warming up the stream back to the operating temperature. Different designs of submerged capillary modules were realized and tested by immersing the capillary fibers directly into the hot feed tank (1 g/L NaCl) while recirculating the distillate inside their lumen [6]. Experiments were carried out by changing the type of membrane (material, pore size, inner diameter, thickness) as well as its length and the module packing density. A process efficiency similar to conventional capillary modules was obtained.

The investigation of the MD performance for treating bio-feeds is an important step to evaluate its potential in the field, where relatively low temperatures must be used. Bioethanol is generated, starting from renewable materials through saccharification and fermentation processes, and its production is linked to the sugar content in the prehydrolyzates. Sweep Gas Membrane Distillation (SGMD) was used to concentrate glucose syrup and the parameters that mostly affected the permeate flux were identified [7]. Feed temperature and sweep gas flow rate had the most significant impact; however, in all tests, 99% of glucose rejection was registered, confirming the efficiency of the process for obtaining concentrated glucose streams.

The potential of DCMD to treat at low-temperature feeds containing urea (urine and human plasma ultrafiltrate) was also presented [8]. Complete rejections for urea were registered, allowing the production of concentrate sources for urea/nutrients recovery, together with purified water as a distillate. As a case study, modules of different sizes and properties were tested to improve the permeate production during the treatment of the human plasma ultrafiltrate, and inputs for further research were identified.

The integration of different membrane units is often a means to improve the overall performance of the process, overcoming the limits of single units. An integrated membrane system consisting of Ultrafiltration (UF), Reverse Osmosis (RO) and DCMD was studied for the treatment of the wastewater coming from a flue gas desulfurization (FGD) plant [9]. A total water recovery of 94% was obtained, with a reduction in the volume of wastewater to be disposed. Moreover, the MD permeate stream was suitable to be reused in the power plant.

The evaluation of MD at pilot scale is a crucial aspect for its implementation at the industrial level. SGMD was investigated and found to concentrate (from 30% to 50%) in a commercial module of 1.2 m^2^ membrane area aqueous solutions of 1,3-dimethyl-2-imidazolidinone (DMI), a solvent used in different fields, like organic reactions and polymer manufacturing [10]. DMI losses were below 1% of the evaporated flux and more than 99.2% of DMI was recovered in the concentrated solution. Based on the obtained results, empirical models were applied to scale-up the process, evidencing the need for improvements for industrial application.

The production of a permeate with a constant quality in time is essential for the large-scale application of MD. Three commercial spiral wound modules with an area ranging from 7.2 to 24 m^2^ were used to evaluate the permeate quality during desalination (feeds of 0.6–2.4 M) [11]. During more than 300 h of test, all modules led to a good permeate quality, with rejections higher than 99%. However, a poor permeate quality was obtained when the process was restarted (intermittent operation), probably due to the presence of membrane pinholes. Further investigations are needed to better understand the behaviour, especially at high-salinity feeds.

The presented papers clearly show the efforts and trends in Membrane Distillation research and development. 

My sincere thanks to all the contributors, who make this Special Issue successful.
